# Associations of Serum Homocysteine with Bone Mineral Density and Osteoporosis Incidence in Chinese Middle-Aged and Older Adults: A Retrospective Cohort Study

**DOI:** 10.3390/nu17010192

**Published:** 2025-01-06

**Authors:** Jiupeng Zou, Mi Shu, Jiedong Chen, Maierhaba Wusiman, Jialu Ye, Sishi Yang, Si Chen, Zihui Huang, Bixia Huang, Aiping Fang, Huilian Zhu

**Affiliations:** 1 Guangdong Provincial Key Laboratory of Food, Nutrition and Health, School of Public Health, Sun Yat-sen University, Guangzhou 510080, China; zoujp7@mail2.sysu.edu.cn (J.Z.); chenjd36@mail2.sysu.edu.cn (J.C.); mehbwsm@mail2.sysu.edu.cn (M.W.); yejlu@mail2.sysu.edu.cn (J.Y.); chens37@mail2.sysu.edu.cn (S.C.); huangzh85@mail2.sysu.edu.cn (Z.H.); huangbix@mail3.sysu.edu.cn (B.H.); fangaip@mail.sysu.edu.cn (A.F.); 2 Guangzhou Shiyuan Health Management Company Limited, Guangzhou 510000, China; shumi@cvte.com (M.S.); yangsishi@cvte.com (S.Y.)

**Keywords:** homocysteine, osteoporosis, risk factor, bone mineral density

## Abstract

Objective: This research investigated the prospective association of serum homocysteine with lumbar bone mineral density (BMD) and the risk of osteoporosis in the Chinese population. Methods: In this cohort, 2551 Chinese individuals aged ≥50 years underwent annual health examinations. Among them, 2551, 1549, and 926 completed two, three, and more than three examinations, respectively. We used generalized estimating equations to analyze the connection between serum homocysteine and lumbar BMD. Additionally, we assessed the connection between serum homocysteine and the incidence of osteoporosis using Cox proportional hazard models. Subgroup analyses based on covariates were performed to identify important at-risk populations. Results: Participants with higher homocysteine levels showed decreased lumbar BMD compared to those with lower homocysteine levels (*p*-trend < 0.05). Specifically, lumbar BMD decreased by −0.002 (−0.003, −0.001) g/cm^2^ for every standard deviation increase in log-transformed serum homocysteine. Compared to the lowest quartile of homocysteine levels, lumbar BMD decreased by −0.006 (−0.010, −0.002) g/cm^2^ in the highest quartile in men. In smokers, lumbar BMD decreased by −0.007 (−0.012, −0.003) g/cm^2^ in the highest quartile. During the follow-up period, 175 incidences of osteoporosis were recorded. Serum homocysteine was linked to an increased risk of osteoporosis (*p*-trend < 0.05). Furthermore, for every standard deviation rise in log-transformed homocysteine, the HR for osteoporosis was 1.33 (95% CI, 1.12–1.58). Conclusions: Elevated homocysteine levels may be responsible for reduced lumbar BMD in middle-aged and older Chinese people, especially men and smokers. In addition, elevated homocysteine levels may be a risk factor for the development of osteoporosis.

## 1. Introduction

Osteoporosis, affecting over 200 million individuals globally [1], is defined as declining bone mineral density (BMD) and deteriorating bone microarchitecture [2]. This issue is increasingly becoming a concern for global health as it leads to increased fragility fractures, morbidity, mortality, and economic burden [3]. Currently, BMD assessment through dual-energy X-rays is the primary method used to diagnose osteoporosis clinically [4]. Although there are clinically available drugs for osteoporosis [2], there is no cure for osteoporosis. Furthermore, the disease is often under-diagnosed and lacks appropriate treatment [5]. Therefore, determining modifiable risk factors for osteoporosis and effective prevention are presently common subjects in osteoporosis research.

Homocysteine (Hcy) is a byproduct generated in the breakdown of methionine [6], which is influenced by factors such as betaine, folate, and diet, and can be easily altered by dietary interventions [7]. As indicated by other studies [8,9], the elevation of Hcy has direct detrimental effects on bone metabolism. Several studies have shown that elevated Hcy levels may interfere with the cross-linking of collagen. This would affect the bone matrix and reduce bone quality [10,11]. In vitro studies have also found that Hcy induces apoptosis in bone marrow stromal cells, thereby reducing bone formation [12]. In addition, Hcy directly promotes osteoclast formation and activity by increasing intracellular reactive oxygen species (ROS) production, thereby promoting bone resorption [13]. Thus, homocysteine, which can be reduced by nutritional interventions, could be an early modifiable osteoporosis risk factor.

In light of these results, epidemiological investigations have been carried out to investigate the connection between serum Hcy and osteoporosis. A previous study showed that Hcy concentrations had a positive association with risk of hip fracture [14]. However, the findings on determining the link between Hcy and BMD are inconsistent, including inverse [15,16,17,18], mixed [19,20,21], and no association [22,23]. A cross-sectional study conducted in Italy reported a negative correlation between Hcy and total femoral BMD [20]. A report from the OFELY study showed no association between Hcy and bone density [22]. Notably, most of these studies were cross-sectional. In addition, few studies have explored the association of Hcy with the risk of osteoporosis and BMD in the Chinese population. The estimated prevalence of Hyperhomocysteinemia (HHcy) in China is 37.2% [24]. The prevalence of osteoporosis in the Chinese population aged 40 years or older was 5.0% in men and 20.6% in women [25]. Therefore, findings from research conducted in other populations might not be directly applicable to Chinese adults.

In order to bridge this gap, we explore the prospective correlation between serum Hcy levels and lumbar bone mineral density, as well as the risk of osteoporosis within the Chinese population.

## 2. Materials and Methods

### 2.1. Study Participants

In this cohort, 6379 Chinese individuals aged ≥50 years underwent annual health examinations at the Yibicom Health Management Center in Guangzhou, China. Among them, 2551, 1549, and 926 completed two, three, and more than three examinations, respectively, during the seven years from 2016 to 2023.

**Figure 1:**
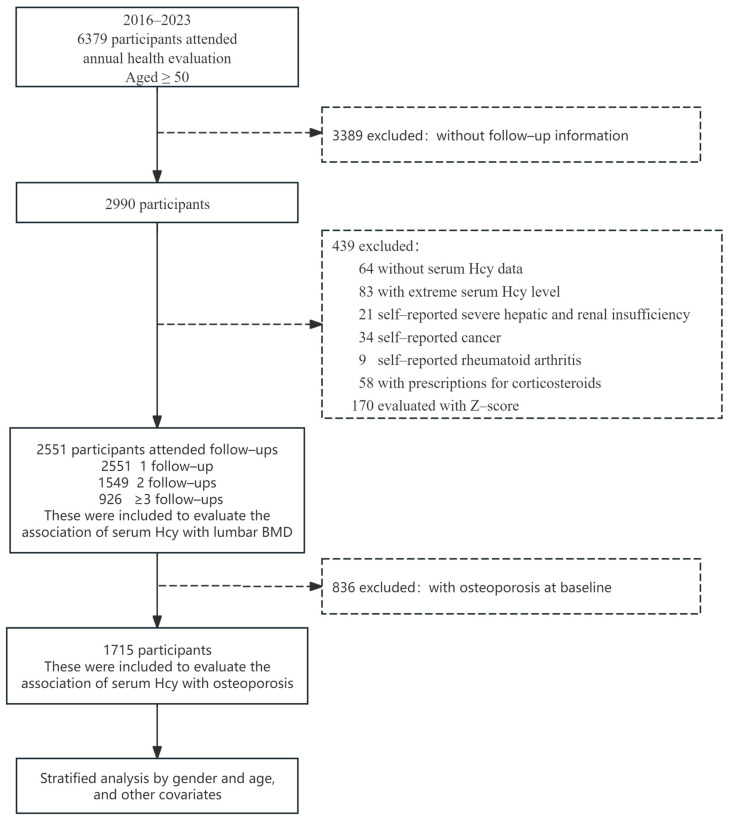
Flowchart of study participants.

Among the 6379 participants at the baseline, there were 3389 participants with no follow-up information. Our predefined criteria included patients (1) without serum Hcy data (*n* = 64); (2) with extreme serum Hcy levels (outside ±3 standard deviations (SD) from the mean concentration) (*n* = 83); (3) with self-reported severe hepatic and renal insufficiency (*n* = 21); (4) with self-reported cancer (*n* = 34); (5) with self-reported rheumatoid arthritis (*n* = 9); (6) with prescriptions for corticosteroids (*n* = 58), and (7) were evaluated with a Z-score (*n* = 170). Because a history of rheumatoid arthritis and corticosteroid use both have potential effects on bone metabolism, this group was excluded. In summary, 439 participants were excluded. Ultimately, we included 2551 participants with baseline Hcy measurements and a minimum of two BMD measurements to explore the association between Hcy levels and BMD. To conduct the analysis on the association between Hcy and incident osteoporosis, individuals diagnosed with osteoporosis at baseline (*n* = 836) were excluded, resulting in 1715 participants being retined for further consideration (Figure 1).

The study protocol received approval from the Public Health Ethics Committee at Sun Yat-sen University (No. 2024-026) on 3 April 2024. The study design, study implementation, data reporting, and dissemination plans did not involve patient or public participation. Since the information was extracted from the medical examination system and de-identified, informed consent was not required.

### 2.2. Data Collection

During the physical examination, data were gathered by trained medical personnel using a structured questionnaire. This survey included comprehensive data on medical history (cardiovascular diseases, hypertension, diabetes, etc.), lifestyle factors (history of drinking and smoking), and demographics (age and gender). Alcohol intake was defined as drinking every day for at least six months in a row, while those who smoked for at least six months or longer were considered smokers.

### 2.3. Physical Examination

In accordance with international standards, physical measures were carried out using an Omron weight scale (model HNH-219, Tokyo, Japan) and an Omron electronic blood pressure monitor (model HBP-9021, Tokyo, Japan). To ensure precision, height and weight were assessed with a precision of 0.1 cm and 0.1 kg, respectively, and participants had to take off their shoes, stand barefoot and upright, and wear a medical gown. Next, their body mass index (BMI) was calculated by dividing their weight (kg) by the square of their height (m^2^). After a five-minute break without consuming any coffee, alcohol, or tobacco, or being emotionally disturbed, we took their blood pressure. Two readings of the right arm were taken, separated by one to two minutes, and the average of the two was recorded.

### 2.4. Biochemical Measurements

Biochemical measurements were conducted at the Yibicom Health Management Center. The laboratory is accredited to ISO 15189 [26] (registration number: CNASMT0691). Following 8–12 h of fasting; then, fasting venous blood samples were collected. Serum creatinine (Cr) was measured via a creatine oxidase assay. Alkaline phosphatase (ALP) was measured via a p-nitrophenylphosphate assay. Serum calcium was assessed using the azo-arsenate method. Serum phosphorus was assessed using phosphomolybdate. Uric acid was measured using the enzyme uricase. Aspartate aminotransferase (AST) was assessed using the nicotinamide adenine dinucleotide (NADH) method, and alanine aminotransferase (ALT) was also assessed using this method. Urea nitrogen was measured using urease, and gamma glutamyltransferase (GGT) using gamma-glutamyl-3-carboxy-4-nitroanilide. Total bilirubin was measured via vanadate oxidation. Fasting blood glucose (FBG) levels were measured using the hexokinase method. Total triglycerides (TG), cholesterol (TC), and high-density lipoprotein cholesterol (HDL-C) were measured by utilizing enzymatic methods. The inter-assay coefficient of variation (CV) of serum Hcy concentration was determined to be 4.2% using a cyclic enzyme assay. Calibration was performed using a NineStrong reagent calibrator(Beijing Strong Biotechnologies, Inc., Beijing, China), which is traceable to the international reference material NIST SRM 1955, using the Spline method. These assays were performed on an Abbott i2000SR chemiluminescent immunoassay analyzer (Abbott, Green Oaks, IL, USA). The coefficients of variation for these tests were consistently less than 6.7%.

### 2.5. BMD Measurements

The areal BMD (g/cm^2^) of the lumbar region was measured through dual-energy X-ray absorptiometry (DXA) with the HOLOGIC DISCOVERY DXA (Discovery A, Hologic Inc., Marlborough, MA, USA). Repeated measurements were carried out with the same instruments as those utilized during the initial assessments. It was found that the machine’s in vivo precision was 1.00%. T-scores were calculated by comparing the BMD of the subjects with the peak BMD of healthy 30-year-old people of the same sex. Based on the WHO standards, a T-score less than or equal to −2.5 indicates osteoporosis, a T-score within the range of −2.5 to −1 indicates low BMD (bone loss), and a T-score greater than or equal to −1 is considered normal BMD [27].

### 2.6. Statistical Analysis

Based on the sex-specific quartiles of serum Hcy levels, participants were split into four groups. The median (interquartile range, or IQR) was used to report continuous data. For categorical data, frequency was expressed as a percentage (%). To assess distinction in baseline characteristics among serum Hcy quartiles, Kruskal–Wallis tests were employed for continuous variables, while chi-square tests were utilized for categorical variables.

Generalized estimating equations (GEEs) were applied to investigate the longitudinal connections between serum Hcy and lumbar BMD. Subgroup analyses based on covariates were performed to identify important at-risk populations. We determined the regression coefficient (β) for serum Hcy levels (quartiles and continuous natural log-transformed) on lumbar BMD. We performed a linear trend analysis by treating each Hcy quartile as a continuous variable within the models. We employed Cox proportional hazards models to assess the relationship between serum Hcy quartiles and the incidence of osteoporosis, using the lowest quartile group as the reference. The hazard ratios (HRs) and 95% confidence intervals (95% CIs) were presented. The selection of covariates was based on the definition of confounders and previous literature with similar research objectives [28,29,30]. The final model covariates were age, gender, baseline BMD, baseline T-score, alcohol drinker (yes or no), smoker (yes or no), BMI, hypertension (yes or no), serum phosphorus, serum calcium, Cr, ALP, TG, TC, and HDL-C.

To determine if there were differences in the relationships between the quartiles of serum Hcy and osteoporosis risk, we conducted stratified analyses in subgroups based on a number of characteristics, including age, gender, smoker, alcohol drinking, and BMI (<25, ≥25 kg/m^2^). Additionally, by including multiplicative factors in the multivariable-adjusted model, we employed the likelihood ratio test to investigate the relationships between serum Hcy and covariates.

GEEs and Cox proportional hazards models were performed using IBM SPSS Statistics 26.0 (SPSS Inc., Chicago, IL, USA). Baseline characterization tables were produced using R (software version 4.3.2). All statistical analyses were conducted using a two-sided approach, with the significance level set at *p* < 0.05.

## 3. Results

### 3.1. Baseline Characteristics

At the baseline, the median (IQR) age of the 2551 individuals was 55.0 (53.0–60.0) years, with a maximum of 79 years and a minimum of 50 years. Of these, 1322 (51.8%) were female. The median age (IQR) of the women was 55.0 (53.0–59.0) years, with a maximum of 79 years and a minimum of 50 years. The median age (IQR) of the men was 56.0 (53.0–60.0) years, with a maximum of 78 years and a minimum of 50 years. The median (IQR) concentration of Hcy was 11.0 (9.3–13.4) μmol/L. Table 1 presents the baseline characteristics according to the Hcy quartiles. In contrast to the lowest quartile, participants in the higher serum Hcy quartile had higher BMI, SBP, DBP, Cr, ALP, prevalences of hypertension, and uric acid, and lower HDL-C, TC, ALT, GGT, and FBG.

In comparison to non-osteoporotic participants, those with osteoporosis at baseline exhibited advanced age, elevated levels of ALP, and increased serum phosphorus (Table S1).

### 3.2. Longitudinal Correlation Between Serum Hcy and Lumbar BMD—Generalized Estimating Equations

Table 2 presents the prospective relationship between baseline serum Hcy and lumbar BMD. In contrast to the lowest quartile group, individuals who had elevated Hcy levels exhibited reduced lumbar BMD and T-scores after adjusting for age, gender, follow-up time, baseline BMD/T-score, alcohol drinking, smoking, BMI, hypertension, serum phosphorus, serum calcium, Cr, ALP, TG, TC, and HDL-C (Model 3, lumbar BMD: *p*-trend = 0.006; T-score: *p*-trend = 0.005). A one-SD increase in log-transformed Hcy levels was linked to a −0.002 (−0.003, −0.001) g/cm^2^ decrease in lumbar BMD and a −0.015 (−0.024, −0.006) decrease in T-score.

Subgroup analyses showed inconsistent associations between serum Hcy levels and decreased lumbar BMD across gender and smoking status (*p*-interaction < 0.05). In men, BMD decreased by −0.006 (−0.010, −0.002) g/cm^2^ in the highest quartile compared to the lowest quartile of Hcy levels. In the smoking population, there was a −0.007 (−0.012, −0.003) g/cm^2^ decrease in BMD in the highest quartile compared to the lowest quartile of Hcy levels. The other stratified variables did not produce a significant modification in the correlation between serum Hcy levels and lumbar BMD (all *p*-interactions > 0.05) (Table 3).

### 3.3. Relationship Between Serum Hcy and Risk of Osteoporosis—Cox Proportional Hazards Models

Excluding participants with osteoporosis at baseline (*n* = 836) from the Hcy–osteoporosis correlation analysis resulted in a subset of 1715 participants. Throughout the follow-up, 175 osteoporosis cases were detected. After adjusting for all possible influences in Model 3, participants with elevated Hcy levels exhibited an increased risk of osteoporosis in comparison to those in the lowest quartile group (Model 3, *p*-trend = 0.034). For each SD increase in log-transformed Hcy, the HR for osteoporosis was 1.33 (95% CI, 1.12–1.58) (Table 4).

### 3.4. Stratified Analyses

Table S2 presents the results of the analyses stratified by age (≤55.5 [median], >55.5 years), gender (men, women), smoking status (yes, no), alcohol consumption (yes, no), and BMI (<25, ≥25 kg/m^2^). The stratified variables did not produce a significant modification in the correlation between serum Hcy levels and the risk of osteoporosis (all *p*-interactions > 0.05).

## 4. Discussion

In this longitudinal investigation involving 2551 participants, our findings revealed a negative correlation between serum Hcy and lumbar BMD (*p*-trend = 0.006), despite adjusting for potential confounding factors. In contrast to the lowest quartile group, individuals with elevated Hcy levels exhibited an increased risk of osteoporosis (*p*-trend = 0.034). To our understanding, this study represents the first longitudinal investigation into the association of serum Hcy with lumbar BMD and the risk of osteoporosis within the Chinese population.

We observed an inverse correlation between serum Hcy and lumbar BMD, which aligns with former research findings. An investigation of combined data from a cross-sectional study involving two cohorts from the B-PROOF study (*n* = 1227) and the Rotterdam studies (RS-I (*n* = 2850) and RS-II (*n* = 2023)) revealed noteworthy correlations between Hcy and BMD in both the femoral neck (B = −0.032, *p* = 0.010) and lumbar (B = −0.098, *p* = 0.021) regions [31]. In a cross-sectional analysis involving 293 young Indian women, the findings indicated an inverse relationship between Hcy and BMD, following adjustments for age, dietary intake, and BMI [28]. Interestingly, the effect of Hcy on BMD was only observed in men. This may be due to the sudden drop in estrogen levels in women during menopause. Studies have shown that estrogen deficiency not only affects osteoblasts [32], but also implicates various indirect mechanisms, such as ROS, cytokines, and growth factors [33], leading to accelerated bone conversion and bone loss. In the present study, we hypothesized that the effects of the dramatic changes induced by estrogen deficiency in postmenopausal women may be sufficient to counteract the effects of Hcy. And in contrast to women, most men do not experience sudden hypogonadism with age. Therefore, further studies on the relationship between sex hormone–Hcy interactions and bone metabolism are needed and may help to explain sex differences in the association of Hcy and low bone mass. In addition, we found a significant effect of Hcy on reduced BMD in smokers. It has previously been shown that smoking affects bone health and may lead to reduced BMD by affecting hormone levels, blood circulation, and bone cell function [34]. This effect may be superimposed on or interact with the effects of Hcy, making the relationship more pronounced in smokers. Further research is needed to better understand this mechanism. On the other hand, we observed that individuals with higher Hcy concentrations had a higher risk of osteoporosis compared with the lowest quartile group (*p*-trend = 0.034). For each SD increase in log-transformed Hcy, the HR for osteoporosis was 1.33 (95% CI, 1.12–1.58). These results are similar to those of a previous study conducted among postmenopausal individuals diagnosed with type 2 diabetes. For every 5.0 μmol/L increment, serum Hcy was associated with osteoporosis (OR = 1.42 (1.07–1.96), *p* = 0.027) [35]. A study that recruited 188 healthy Moroccan postmenopausal women also found an effect of serum Hcy on osteoporosis [36]. Therefore, we concluded that Hcy is a significant predictor of lumbar BMD reduction and osteoporosis risk in the middle-aged and older Chinese populations. Given the results derived from this paper and previous related studies, a healthy diet, nutraceuticals, and/or appropriate physical activity may be further strategies to improve bone structure and function in Chinese middle-aged and elderly people [37,38].

However, previous research has indicated that Hcy is unrelated to BMD. For example, a study among young (mean age 26), healthy, vegetarian Indian adults showed no correlation between Hcy and BMD [39]. A study conducted on kidney transplant recipients (KTR) showed no correlation between serum Hcy levels and BMD [40]. Findings from a study involving 131 healthy women aged 45–65 years in Croatia similarly demonstrated that Hcy is not associated with BMD [23]. Most of these studies are cross-sectional [39,40], with smaller sample sizes and less focus on relatively healthy populations. And they did not involve Chinese populations. There are two possible reasons for these conflicting findings. On the one hand, they may be due to differences between studies, including differences in study design, study size, and participant selection (including age and gender). On the other hand, they may be due to differences in dietary structure. For example, the ‘Oriental diet’ contains higher levels of isoflavones in soya and soya-derived foods, which have an effect on bone structure and function [41,42].

Experimental studies have indicated that serum Hcy could impact the bone matrix and reduce bone quality by impeding the cross-linking of collagen fibers and disrupting the formation of a collagen network structure [10,11]. Elevated Hcy levels have been associated with reduced osteocalcin expression and heightened osteopontin expression, leading to the disruption of normal osteoblast function, ultimately resulting in osteoporosis [43]. Additionally, in laboratory studies, it has been observed that Hcy triggers apoptosis in bone marrow stromal cells through the reactive oxygen species (ROS)-mediated mitochondrial pathway and NF-kappa B activation. Simultaneously, it fosters bone resorption by enhancing p38 mitogen-activated protein kinase (MAPK) activity and promoting the generation of intracellular ROS in osteoclasts [12,13]. It has also been found that the effect of Hcy on bone loss may be due to the net effect of a gradual increase in RANKL and a decrease in OPG, which leads to an increase in osteoclastogenesis [44]. The prospective correlation between serum Hcy levels and lumbar bone mineral density, as well as the risk of osteoporosis, may be due to these aspects.

This study exhibits several notable strengths. Firstly, this is the first longitudinal cohort study of Hcy and lumbar BMD in the Chinese population. Secondly, with participants from a wide range of genders, ages, and geographic locations around the country, our study had a sizable and varied sample size. This improves our findings’ representativeness and makes them easier to generalize. Thirdly, to comprehensively explore the exclusive physiological impacts of Hcy, we applied rigorous exclusion criteria and controlled for variables recognized to influence bone metabolism.

Inevitably, this research presents some limitations. Firstly, as a retrospective cohort study, data collection is susceptible to missing data, measurement errors, and inaccuracies. Secondly, while we made effort to consider numerous confounding factors, we cannot discount the potential for the observed correlations to be attributed to other variables impacting Hcy [45,46,47] and/or BMD [48], including 25-hydroxyvitamin D levels, dietary patterns, gut microbiome composition, exercising regularly, etc. Therefore, the association between Hcy and decreased BMD observed in this study may be due to poor nutritional status. However, our cohort consisted mainly of relatively young and healthy subjects who were unlikely to have nutritional deficiencies, which means that our results may provide additional evidence for a causal relationship between Hcy and decreased BMD. Thirdly, this study only involved measurements of BMD in the lumbar region, and further research will be needed on BMD in other areas of the body. Finally, all of the participants in this study were Chinese. Therefore, we cannot guarantee that our findings will hold true for different populations.

## 5. Conclusions

In summary, elevated homocysteine levels may be responsible for the reduced lumbar BMD in middle-aged and older Chinese people, especially men and smokers. In addition, elevated homocysteine levels may be a risk factor for the development of osteoporosis. The results presented in this research may have clinical significance for the prevention of osteoporosis and the reduction in lumbar BMD in the Chinese population. Further interventional studies are needed to confirm the preventive effect of lowering Hcy levels on osteoporosis and BMD loss.

## Figures and Tables

**Table 1:** Baseline characteristics by sex-specific quartiles of serum homocysteine (*n* = 2551).

	Sex-Specific Quartiles of Serum Homocysteine				*p*-Value
	Q1 (*n* = 638)	Q2 (*n* = 637)	Q3 (*n* = 639)	Q4 (*n* = 637)	
Age, years	55.0 (52.0, 59.0)	55.0 (53.0, 60.0)	55.0 (53.0, 60.0)	57.0 (53.0, 61.0)	<0.001
Women, *n* (%)	331 (51.9)	330 (51.8)	331 (51.8)	330 (51.8)	1
Homocysteine, μmol/L	8.2 (7.4, 9.3)	10.0 (9.3, 11.3)	11.9 (10.8, 13.2)	15.8 (13.7, 18.5)	<0.001
Lumbar BMD, g/cm^2^	0.84 (0.76, 0.95)	0.85 (0.75, 0.94)	0.85 (0.76, 0.96)	0.86 (0.77, 0.95)	0.257
Lumbar T-score	−2.1 (−2.8, −1.1)	−2.1 (−2.8, −1.2)	−2.0 (−2.8, −1.0)	−1.9 (−2.7, −1.0)	0.182
BMI, kg/m^2^	23.6 (21.6, 25.8)	23.5 (21.7, 25.4)	24.1 (22.2, 26.4)	24.3 (22.2, 26.1)	<0.001
Smoker, *n* (%)	208 (32.6)	196 (30.8)	219 (34.3)	221 (34.7)	0.43
Alcohol drinker, *n* (%)	175 (27.4)	165 (25.9)	171 (26.8)	172 (27.0)	0.94
Hypertension, *n* (%)	104 (16.3)	119 (18.7)	138 (21.6)	175 (27.5)	<0.001
Diabetes, *n* (%)	43 (6.7)	43 (6.8)	40 (6.3)	50 (7.8)	0.718
Cardiovascular diseases, *n* (%)	17 (2.7)	21 (3.3)	26 (4.1)	32 (5.0)	0.141
SBP, mmHg	128.0 (116.0, 142.0)	129.0 (117.0, 142.0)	131.0 (118.0, 145.0)	133.0 (120.0, 149.0)	<0.001
DBP, mmHg	76.0 (68.0, 85.0)	77.0 (68.0, 85.0)	78.0 (69.5, 86.0)	78.0 (70.0, 86.0)	0.017
Serum calcium, mmol/L	2.3 (2.3, 2.4)	2.3 (2.3, 2.4)	2.4 (2.3, 2.4)	2.3 (2.3, 2.4)	0.604
Serum phosphorus, mmol/L	1.1 (1.0, 1.2)	1.2 (1.0, 1.3)	1.1 (1.0, 1.2)	1.1 (1.0, 1.2)	0.326
Uric acid, μmol/L	314.0 (267.0, 368.0)	335.0 (277.0, 393.0)	338.0 (286.0, 405.0)	341.0 (286.0, 412.0)	<0.001
FBG, mmol/L	5.52 (5.23, 5.94)	5.53 (5.20, 5.95)	5.47 (5.17, 5.95)	5.46 (5.12, 5.90)	0.017
TG, mmol/L	1.26 (0.95, 1.82)	1.31 (0.90, 1.95)	1.32 (0.96, 1.96)	1.34 (0.97, 1.92)	0.349
TC, mmol/L	5.30 (4.74, 5.95)	5.27 (4.68, 5.95)	5.20 (4.64, 5.88)	5.02 (4.43, 5.72)	<0.001
HDL-C, mmol/L	1.29 (1.11, 1.52)	1.28 (1.09, 1.49)	1.25 (1.07, 1.48)	1.21 (1.02, 1.44)	<0.001
ALP, IU/L	76.0 (64.0, 89.0)	77.0 (65.0, 90.0)	77.0 (67.0, 92.0)	78.0 (67.0, 91.0)	0.024
ALT, IU/L	21.0 (16.0, 28.0)	21.0 (16.0, 29.0)	21.0 (15.0, 30.0)	20.0 (15.0, 26.0)	0.01
AST, IU/L	21.0 (18.0, 25.0)	21.0 (18.0, 25.0)	21.0 (18.0, 25.0)	21.0 (18.0, 25.0)	0.452
Cr, μmol/L	62.0 (53.0, 74.0)	65.0 (55.0, 75.0)	67.0 (57.0, 78.0)	68.0 (58.0, 82.0)	<0.001
BUN, mmol/L	4.9 (4.2, 5.8)	4.9 (4.1, 5.9)	4.9 (4.1, 6.0)	5.2 (4.1, 6.3)	0.126
GGT, IU/L	21.8 (15.9, 32.7)	22.3 (16.2, 37.0)	22.0 (15.8, 36.0)	20.4 (14.5, 30.7)	0.003
Total bilirubin, μmol/L	11.8 (9.6, 14.3)	11.4 (9.4, 14.3)	11.5 (9.2, 14.3)	11.4 (9.0, 14.3)	0.451

Abbreviations: Q1, first quartile; Q2, second quartile; Q3, third quartile; Q4, fourth quartile; BMD, bone mineral density; BMI, body mass index; SBP, systolic blood pressure; DBP, diastolic blood pressure; FBG, fasting blood glucose; TG, triglycerides; TC, total cholesterol; HDL-C, high-density lipoprotein cholesterol; ALP, alkaline phosphatase; ALT, alanine aminotransferase; AST, aspartate aminotransferase; Cr, creatinine; BUN, blood urea nitrogen; GGT, gamma glutamyltransferase. The data are shown as median (IQR) for continuous variables and as frequency (%) for categorical variables.

**Table 2:** The prospective association of serum Hcy with lumbar BMD and T-score (*n* = 2551).

	Sex-Specific Quartiles of Serum Homocysteine					
	Q1 (*n* = 638)	Q2 (*n* = 637)	Q3 (*n* = 639)	Q4 (*n* = 637)	*p*-Trend *	Per-SD Increase in lg (Hcy Levels)
Lumbar BMD						
Model 1	Ref.	−0.002 (−0.005, 0.001)	−0.002 (−0.004, 0.001)	−0.003 (−0.006, −0.001)	0.016	−0.001 (−0.002, −0.0005)
Model 2	Ref.	−0.002 (−0.005, 0.001)	−0.002 (−0.005, 0.001)	−0.004 (−0.006, −0.001)	0.007	−0.002 (−0.003, −0.001)
Model 3	Ref.	−0.002 (−0.005, 0.0005)	−0.002 (−0.005, 0.001)	−0.004 (−0.007, −0.001)	0.006	−0.002 (−0.003, −0.001)
T-score						
Model 1	Ref.	−0.018 (−0.043, 0.006)	−0.015 (−0.039, 0.009)	−0.032 (−0.054, −0.009)	0.015	−0.013 (−0.022, −0.004)
Model 2	Ref.	−0.018 (−0.042, 0.006)	−0.017 (−0.041, 0.007)	−0.034 (−0.057, −0.011)	0.006	−0.014 (−0.023, −0.006)
Model 3	Ref.	−0.020 (−0.044, 0.005)	−0.018 (−0.042, 0.006)	−0.037 (−0.060, −0.013)	0.005	−0.015 (−0.024, −0.006)

Generalized estimating equations were used to calculate Beta-coefficients and 95% CIs of lumbar BMD and T-score in quartiles 2–4 (vs. 1) of serum homocysteine. Abbreviations: CI, confidence interval; Ref., reference. * *p* for trend was computed by considering sex-specific quartiles of serum homocysteine as continuous values in generalized estimating equations. Model 1: adjusted for age, gender, baseline BMD/T-score, and follow-up time. Model 2: model 1 further adjusted for smoking, alcohol drinking, BMI, and hypertension. Model 3: model 2 further adjusted for serum phosphorus, serum calcium, Cr, ALP, TG, TC, and HDL-C.

**Table 3:** Stratified Beta-coefficients and 95% CIs for lumbar BMD and T-score according to baseline serum homocysteine levels (quartiles) (*n* = 2551).

	*n*	Sex-Specific Quartiles of Serum Homocysteine				*p*-Interaction ^a^
		Q1	Q2	Q3	Q4	
Gender						
Lumbar BMD						0.022
Men	1229	Ref.	−0.002 (−0.005, 0.002)	−0.003 (−0.007, 0.001)	−0.006 (−0.010, −0.002)	
Women	1322	Ref.	−0.004 (−0.007, 0.0003)	−0.002 (−0.005, 0.002)	−0.002 (−0.006, 0.001)	
T-score						0.014
Men	1229	Ref.	−0.014 (−0.048, 0.020)	−0.030 (−0.064, 0.004)	−0.060 (−0.095, −0.025)	
Women	1322	Ref.	−0.032 (−0.064, 0.003)	−0.015 (−0.048, 0.018)	−0.017 (−0.049, 0.014)	
Age, years						
Lumbar BMD						0.475
≤55	1290	Ref.	−0.002 (−0.005, 0.002)	−0.001 (−0.005, 0.002)	−0.002 (−0.006, 0.002)	
>55	1261	Ref.	−0.003 (−0.007, 0.001)	−0.003 (−0.006, 0.001)	−0.005 (−0.009, −0.002)	
T-score						0.483
≤55	1290	Ref.	−0.015 (−0.048, 0.017)	−0.013 (−0.047, 0.021)	−0.019 (−0.055, 0.016)	
>55	1261	Ref.	−0.025 (−0.061, 0.010)	−0.024 (−0.057, 0.009)	−0.048 (−0.079, −0.017)	
Smoker						
Lumbar BMD						0.040
No	1707	Ref.	−0.003 (−0.006, 0.001)	−0.001 (−0.005, 0.002)	−0.002 (−0.005, 0.001)	
Yes	844	Ref.	−0.002 (−0.007, 0.002)	−0.003 (−0.008, 0.001)	−0.007 (−0.012, −0.003)	
T-score						0.025
No	1707	Ref.	−0.024 (−0.052, 0.005)	−0.012 (−0.042, 0.019)	−0.018 (−0.046, 0.011)	
Yes	844	Ref.	−0.018 (−0.060, 0.023)	−0.033 (−0.071, 0.005)	−0.070 (−0.113, −0.026)	
Alcohol drinker						
Lumbar BMD						0.486
No	1868	Ref.	−0.002 (−0.005, 0.001)	−0.001 (−0.005, 0.002)	−0.003 (−0.006, −0.0002)	
Yes	683	Ref.	−0.004 (−0.009, 0.001)	−0.004 (−0.009, 0.001)	−0.006 (−0.012, −0.001)	
T-score						0.371
No	1868	Ref.	−0.019 (−0.046, 0.008)	−0.013 (−0.041, 0.015)	−0.026 (−0.052, 0.0003)	
Yes	683	Ref.	−0.030 (−0.077, 0.018)	−0.036 (−0.080, 0.007)	−0.062 (−0.113, −0.011)	
BMI, kg/m^2^						
Lumbar BMD						0.422
BMI < 25	1639	Ref.	−0.004 (−0.007, −0.0004)	−0.001 (−0.004, 0.002)	−0.003 (−0.006, 0.0003)	
BMI ≥ 25	912	Ref.	0.001 (−0.004, 0.006)	−0.004 (−0.009, 0.001)	−0.005 (−0.009, 0.0001)	
T-score						0.446
BMI < 25	1639	Ref.	−0.032 (−0.061, −0.003)	−0.008 (−0.036, 0.020)	−0.027 (−0.055, 0.001)	
BMI ≥ 25	912	Ref.	0.007 (−0.036, 0.050)	−0.038 (−0.082, 0.007)	−0.043 (−0.087, 0.0003)	

^a^ Interactions between serum homocysteine and the covariates were tested via the likelihood ratio test using the multiplicative interaction term. In the multivariable-adjusted model, the confounding factors included age, gender, baseline lumbar BMD, baseline T-score, alcohol drinking, smoking, BMI, hypertension, serum phosphorus, serum calcium, Cr, ALP, TG, TC, and HDL-C. The median of age was the cut-off point.

**Table 4:** HRs and 95% CIs for incident osteoporosis according to quartiles of baseline serum homocysteine (*n* = 1715).

	Sex-Specific Quartiles of Serum Homocysteine					
	Q1 (*n* = 419)	Q2 (*n* = 426)	Q3 (*n* = 432)	Q4 (*n* = 438)	*p*-Trend *	Per-SD Increase in lg (Hcy Levels)
Median (IQR), μmol/L	8.20 (7.41, 9.42)	10.65 (9.40, 11.41)	12.40 (10.86, 13.29)	16.03 (14.12, 18.62)		
Case, *n*	37	45	50	43		
Model 1	Reference	1.33 (0.86, 2.06)	1.42 (0.92, 2.18)	1.80 (1.14, 2.83)	0.012	1.34 (1.14, 1.57)
Model 2	Reference	1.32 (0.85, 2.06)	1.43 (0.93, 2.20)	1.76 (1.11, 2.79)	0.015	1.34 (1.13, 1.58)
Model 3	Reference	1.31 (0.83, 2.06)	1.37 (0.87, 2.14)	1.70 (1.05, 2.76)	0.034	1.33 (1.12, 1.58)

Cox proportional hazards models were employed to determine HRs and 95% CIs of osteoporosis incidence across quartiles 2–4 (compared to quartile 1) of serum homocysteine. Abbreviations: HR, hazard ratio; CI, confidence interval; Ref., reference. In Cox proportional hazards models, * *p* for trend was computed by considering sex-specific quartiles of serum homocysteine as continuous values. Model 1: adjusted for age, gender, and baseline T-score. Model 2: model 1 further adjusted for smoking, alcohol drinking, BMI, and hypertension. Model 3: model 2 further adjusted for serum phosphorus, serum calcium, Cr, ALP, TG, TC, and HDL-C.

## Data Availability

This study’s original contributions can be found within the article and its Supplementary Materials. For further information, please contact the corresponding author. The data are not publicly available due to privacy reasons.

## Supplementary Materials

The following supporting information can be downloaded at: https://www.mdpi.com/article/10.3390/nu17010192/s1, Table S1: Baseline characteristics of osteoporosis and nonosteoporosis participants; Table S2: Stratified HRs and 95% CIs for incident osteoporosis according to baseline serum homocysteine levels (quartiles).
