# Supplementary material for: Associations of Serum Homocysteine with Bone Mineral Density and Osteoporosis Incidence in Chinese Middle-Aged and Older Adults: A Retrospective Cohort Study
Source: Nutrients. 2025 Jan 6;17(1):192. doi: 10.3390/nu17010192 (PMC11723229; doi:10.3390/nu17010192)
Supplement: Supplementary file 1 [file nutrients-17-00192-s001.zip › nutrients-3355175-supplementary.pdf]

Supplementary Materials

Associations of Serum Homocysteine with Bone Mineral Density and Osteoporosis Incidence in Chinese Middle-Aged and Older Adults: A Retrospective Cohort Study

Jiupeng Zou [1,†], Mi Shu [2,†], Jiedong Chen [1], Maierhaba Wusiman [1], Jialu Ye [1], Sishi Yang [2], Si Chen [1], Zihui Huang [1], Bixia Huang [1], Aiping Fang [1] and Huilian Zhu [1,*]

Supplemental Table S1. Baseline characteristics of osteoporosis and nonosteoporosis participants.

Supplemental Table S2. Stratified HRs and 95% CIs for incident osteoporosis according to baseline serum homocysteine levels (quartiles).

Supplemental Table S1. Baseline characteristics of osteoporosis and nonosteoporosis participants

	Osteoporosis (n = 836)	Nonosteoporosis (n = 1715)	*P*-Value
Age, years	57.0 (54.0, 61.0)	55.0 (52.0, 59.0)	<0.001
Women, n (%)	509 (60.9)	813 (47.4)	<0.001
Homocysteine, μmol/L	10.6 (9.0, 13.1)	11.2 (9.4, 13.5)	<0.001
Lumbar BMD, g/cm^2	0.72 (0.67, 0.76)	0.91 (0.85, 1.00)	<0.001
Lumbar T-score	-3.1 (-3.5, -2.8)	-1.5 (-2.0, -0.7)	<0.001
BMI, kg/m^2	23.0 (21.0, 24.9)	24.4 (22.3, 26.3)	<0.001
Smoker, n (%)	231 (27.6)	613 (35.7)	<0.001
Alcohol drinker, n (%)	177 (21.2)	506 (29.5)	<0.001
Hypertension, n (%)	160 (19.1)	376 (21.9)	0.117
Diabetes, n (%)	38 (4.5)	138 (8.0)	0.001
Cardiovascular diseases, n (%)	38 (4.5)	58 (3.4)	0.181
SBP, mmHg	129.0 (117.0, 143.0)	130.0 (118.0, 145.0)	0.019
DBP, mmHg	76.0 (67.0, 83.0)	78.0 (70.0, 86.0)	<0.001
Serum calcium, mmol/L	2.3 (2.3, 2.4)	2.3 (2.3, 2.4)	0.257
Serum phosphorus, mmol/L	1.2 (1.0, 1.3)	1.1 (1.0, 1.2)	<0.001
Uric acid, μmol/L	311.0 (266.0, 365.2)	342.0 (285.0, 404.0)	<0.001
FBG, mmol/L	5.4 (5.1, 5.8)	5.5 (5.2, 6.0)	<0.001
TG, mmol/L	1.2 (0.9, 1.7)	1.3 (0.9, 2.0)	<0.001
TC, mmol/L	5.2 (4.6, 5.9)	5.1 (4.6, 5.8)	0.113
HDL-C, mmol/L	1.2 (1.1, 1.5)	1.2 (1.0, 1.4)	<0.001
ALP, IU/L	80.0 (68.7, 95.0)	76.0 (64.0, 89.0)	<0.001
ALT, IU/L	19.0 (15.0, 26.0)	21.0 (16.0, 29.0)	<0.001
AST, IU/L	21.0 (18.0, 25.0)	21.0 (18.0, 25.0)	0.265
Cr, μmol/L	63.0 (54.0, 74.0)	67.0 (57.0, 79.0)	<0.001
BUN, mmol/L	5.0 (4.0, 5.9)	5.0 (4.2, 6.0)	0.087
GGT, IU/L	20.1 (14.7, 30.5)	22.3 (16.2, 35.6)	<0.001
Total bilirubin, μmol/L	11.3 (9.5, 14.0)	11.7 (9.2, 14.4)	0.418

Abbreviations: BMD, bone mineral density; BMI, body mass index; SBP, systolic blood pressure; DBP, diastolic blood pressure; FBG, fasting blood glucose; TG, triglycerides; TC, total cholesterol; HDL-C, high-density lipoprotein cholesterol; ALP, alkaline phosphatase; ALT, alanine aminotransferase; AST, aspartate aminotransferase; Cr, creatinine; BUN, blood urea nitrogen; GGT, gamma glutamyltransferase. Data were presented median (interquartile range) for continuous variables; and frequency (percentage) for categorical variables.

Supplemental Table S2. Stratified HRs and 95% CIs for incident osteoporosis according to baseline serum homocysteine levels (quartiles)

	N	Sex-specific quartiles of serum homocysteine				*P*-interaction [a]
		Q1	Q2	Q3	Q4	
**Gender**						0.732
Men	902	Ref.	1.27 (0.54, 2.96)	0.81 (0.31, 2.07)	1.27 (0.49, 3.28)	
Women	813	Ref.	1.14 (0.65, 2.02)	1.53 (0.90, 2.61)	1.68 (0.92, 3.05)	
**Age, years**						0.769
≤55	931	Ref.	1.21 (0.68, 2.14)	1.16 (0.65, 2.06)	1.61 (0.83, 3.13)	
＞55	784	Ref.	1.32 (0.58, 3.01)	2.04 (0.93, 4.45)	2.17 (0.97, 4.82)	
**Smoker**						0.856
No	1102	Ref.	1.35 (0.80, 2.30)	1.55 (0.92, 2.60)	1.89 (1.08, 3.30)	
Yes	613	Ref.	0.98 (0.36, 2.61)	0.82 (0.30, 2.20)	1.11 (0.39, 3.14)	
**Alcohol drinker**						0.976
No	1209	Ref.	1.28 (0.77, 2.13)	1.44 (0.88, 2.36)	1.80 (1.04, 3.09)	
Yes	506	Ref.	1.12 (0.34, 3.71)	0.74 (0.21, 2.62)	1.32 (0.38, 4.62)	
**BMI, kg/m2**						0.885
BMI＜25	1010	Ref.	1.65 (0.96, 2.84)	1.37 (0.78, 2.42)	1.59 (0.86, 2.93)	
BMI≥25	705	Ref.	1.08 (0.38, 3.01)	2.02 (0.86, 4.69)	2.05 (0.84, 5.00)	

[a] Interactions between serum homocysteine and covariates were tested by the likelihood ratio test using the multiplicative interaction term. In the multivariable-adjusted model, confounding factors including age, gender, baseline T-score, alcohol drinker, smoker, BMI, Hypertension, serum phosphorus, serum calcium, Cr, ALP, TG, TC, and HDL-C. The median of age was cut-off points.
